# Loss of M1 Acetylcholine Receptor-mediated Orexinergic Activity Contributes to Immune Dysfunction in Experimental Sepsis

**DOI:** 10.21203/rs.3.rs-7329263/v1

**Published:** 2025-08-25

**Authors:** Ana Nedeljkovic-Kurepa, Mabel N. Abraham, Tiago D. Fernandes, Omar Yaipen, Mariana R. Brewer, Matthew D. Taylor, Valentin A. Pavlov, Clifford S. Deutschman

**Affiliations:** Northwell Health; Northwell Health; Northwell Health; Northwell Health; Northwell Health; Northwell Health; Northwell Health; Northwell Health

**Keywords:** Sepsis, Orexin, Muscarinic Acetylcholine Receptors, Immunology, cecal ligation and puncture

## Abstract

**Background:**

Sepsis (life-threatening organ dysfunction caused by a dysregulated host response to infection) causes millions of deaths worldwide annually. Sepsis-induced changes in brain regulatory functions remain understudied. Previous work demonstrated that cecal ligation and puncture (CLP, a murine model of sepsis) affected physiologic variables and serum cytokines and hormone levels. Correction of decreased activity in the orexinergic nervous system or administration of the M1 muscarinic acetylcholine receptor (M1mAChR) agonist xanomeline reversed some of these findings. We hypothesized that these **CLP - induced changes resulted, in part, from a loss of M1mAChR-mediated orexinergic nervous system activation**.

**Main Body:**

Xanomeline reversed CLP-induced loss of orexinergic activity and restored physiologic variables and hormone levels to baseline; these corrections were eliminated by addition of the orexin receptor antagonist almorexant. To examine the effects of system reactivation we developed a transgenic mouse whose orexinergic neurons could be depolarized via a Designer Receptor Exclusively Activated by Designer Drugs (DREADD) and its ligand, clozapine-N-oxide (CNO). Orexinergic re-activation or xanomeline administration reversed CLP-induced changes in TNFa and IL-1b levels; almorexant eliminated xanomeline effects. CNO reversed the effects of CLP on serum levels of IL-6 and KC; this effect was not present after xanomeline administration. G-CSF, a colony stimulating factor, was not affected by either CNO or xanomeline. Both orexinergic activation and xanomeline administration reversed CLP-induced increase in the number of splenic macrophages and monocyte-derived dendritic cells (DCs); almorexant did not affect the response to xanomeline. CLP-induced decreases in the numbers of central DCs, CD4^+^ or CD8^+^ T cell numbers in the spleen; this response was not altered by either CNO or xanomeline.

**Conclusion:**

Decreased orexinergic activity mediates some post-CLP immunologic changes, identifying a previously unrecognized proximal pathogenic mechanism in sepsis. Some, but not all, of these changes result from a loss of M1mAChR-mediated stimulation of orexinergic neurons. These findings suggests that disruption of orexin’s central coordinating function is a key, and perhaps causative, component of the dysregulated host response that is the defining characteristic of sepsis.

## Background

Sepsis is defined as “life-threatening organ dysfunction caused by a dysregulated host response to infection” (1). This syndrome afflicts nearly 50 million people worldwide and leads to 11 million deaths each year (2). One key component of the dysregulated host response is an inability of organ systems to interact in a coordinated manner; the result is responses are inappropriate (3, 4). The immune, endocrine and neural systems are directly involved in organ-to-organ interaction (5–11). Most studies seeking to identify and correct the altered modulation of host biology that characterizes sepsis have focused on the immune and endocrine systems. Aberrant neural responses, however, have been investigated less extensively.

Investigations during the last 20 years have revealed that inflammation is modulated by the brain via the vagus nerve (11). These studies also linked basal forebrain cholinergic neurons to vagus nerve-mediated modulation of inflammation, forming one limb of a reflex arc that restrains leukocyte activation and cytokine expression (11–13). Recent work revealed that the centrally-acting M1 muscarinic cholinergic receptor (M1mAChR) agonist xanomeline reversed lipopolysaccharide (LPS) - induced elevations in several cytokines and attenuated changes in splenic immune cell populations (13). Xanomeline also improved survival following cecal ligation and puncture (CLP), the most commonly-used animal model of sepsis, and affected some cytokines and splenic cells not examined in LPS studies (13, 14). Overall, these findings suggest that the brain cholinergic system plays a central role in balanced inflammation and in the dysregulated response that underlies sepsis.

Other investigations into sepsis pathobiology have focused on the orexinergic nervous system of the hypothalamus (6). This system, whose neurons secrete the neurotransmitter orexin (also called hypocretin), has been primarily examined for its contribution to arousal and sleep (15). However, activity in the locus also affects cardiodynamics (16–18), respiration (19–21), fluid balance (22), cognition (23, 24), GI function (25), metabolism (26) and pituitary hormone secretion (27). Sepsis affects each of these processes, including consciousness. Our previous work demonstrated that, at 48hrs post-CLP, only 10% of orexinergic neurons were active, down from approximately 60% at baseline (6). When we stimulated orexinergic receptors with intra-cerebroventricular (ICV) orexin, CLP-induced decreases in heart rate (HR), respiratory rate (RR), temperature (T) and in the secretion of pituitary hormones were no longer present (6). Others have identified bi-directional neural pathways linking the orexinergic centers to the basal forebrain cholinergic system (28–31). We therefore hypothesized that one mechanism mediating the capricious immune responses observed post - CLP was an alteration in the interaction between the orexinergic and M1mAChR-mediated cholinergic systems in the brain.

The results presented here show that a decrease in M1mAChR-mediated stimulation of orexinergic activity contributed to CLP-induced differences in vital signs, serum levels of pituitary hormones and some cytokines, and phenotype expression and responses to activation in some splenic immune cell populations. Other cytokines and phenotypic changes were affected by the orexinergic system in an M1mAChR-independent manner. Restoration of orexinergic activity did not, however, affect the CLP-induced reduction in the activity of basal forebrain cholinergic neurons. These findings indicate that the dysregulated host response in sepsis is the result of changes in the complex interaction between the neural, endocrine and immune systems.

## Results

### M1mAChR stimulation with xanomeline attenuates or reverses CLP-induced decreases in orexinergic activity, T, HR, RR and pituitary hormone levels.

**Figure 1:**
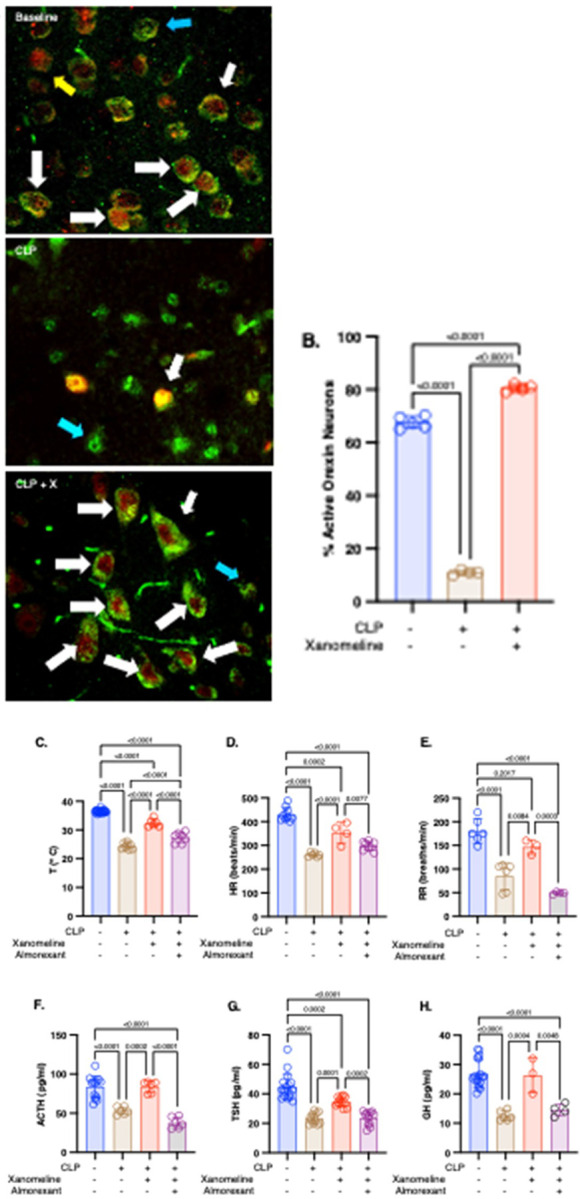
Effects of CLP and treatment of CLP with xanomeline or xanomeline + almorexant on orexinergic activity. Xanomeline administered IP at the time of CLP and at 23 and 47 hrs. post-CLP. Mice were euthanized at 48 hrs. post - CLP. Each individual animal represented by a circle: Blue – baseline. Brown – CLP. Red – CLP + xanomeline. Purple – CLP+ xanomeline + almorexant. Lines; Mean ± SD. P values noted above each significant comparison. **A. Representative stained sections**. 40X magnification. Green stain/blue arrows – orexin. Red stain/yellow arrows) – c-fos, indicative of activity. White arrows indicate cells staining positive for both orexin and c-fos. **B. Quantification of percentage of active orexinergic cells.** Data measured on 10 non-contiguous 40xpowered fields/slide, 1–2 slides/animal. One – way ANOVA. **C– H**: Quantification of parameter indicated on Y-axis. **C. Temperature.** One-way ANOVA. **D. Heart Rate.** One-way ANOVA. **E. Respiratory Rate.** One-way ANOVA. **F. Adrenocorticotropic Hormone (ACTH).** One-way ANOVA. **G. Thyroid Stimulating Hormone (TSH).** One-way ANOVA of log-transformed data. **H. Growth Hormone (GH).** One-way ANOVA.

Previous studies demonstrated that restoring the CLP-induced decrease in orexinergic activity with ICV orexin reversed CLP-induced decreases in T, HR, RR and serum levels of pituitary hormones (6). In addition, treating the post-CLP attenuated M1mAChR-mediated cholinergic system with the centrally acting M1mAChR agonist xanomeline lowered serum levels of some, but not all, cytokine concentrations (14). Therefore, we examined the effects of xanomeline on CLP-induced suppression of orexinergic activity (Figure 1A: representative stained hypothalamic sections; 1B: quantified activity) and on vital signs and pituitary hormone levels (Figures 1 C–H). As in prior studies, at 48 hrs. after CLP all variables were lower than at baseline. When brain M1mAChRs stimulation with xanomeline was added, orexinergic activity was significantly higher than in untreated post-CLP mice. Further, measurements of RR (Figure 1E) and of adrenocorticotropin (ACTH, Figure 1F) and Growth Hormone (GH, Figure 1H) levels in xanomeline - treated mice were indistinguishable from baseline. T (Figure 1C), HR (Figure 1D) and Thyroid Stimulating Hormone (TSH, Figure 1G) levels in treated mice remained significantly lower than at baseline. These findings suggest that the mechanism underlying the CLP-induced loss of orexinergic activity results, in part, from a loss of M1mAChR-induced activation.

### Xanomeline-mediated attenuation/elimination of CLP–induced effects on vital signs and pituitary hormone levels result from reduced M1mAChR-stimulated enhancement of orexinergic activity

CLP-induced decreases in orexinergic activity were eliminated by xanomeline while both ICV orexin (6) and xanomeline (Figure 1C–H) attenuated effects on T, HR, RR and pituitary hormones levels (Figures 1C–G). These findings suggest that post-CLP changes resulted from decreased interaction between M1mAChR-mediated processes and the orexinergic system. To address the possibility that orexinergic neurons are stimulated by M1mAChRs, we treated a subset of the animals receiving xanomeline with almorexant, a non-specific orexin receptor antagonist, at the time of xanomeline administration. The addition of almorexant eliminated the effects of xanomeline on CLP-induced changes in HR, RR, and in serum levels of ACTH, TSH and GH (Figure 1D–H) and attenuated the decrease in T (Figure 1C). These findings indicate that CLP affected T, HR, RR, and serum levels of ACTH, TSH and GH via a mechanism that included a loss of M1mAChR-mediated stimulation of orexinergic activity.

### Orexinergic activation does not affect post-CLP activity in ChAT-expressing basal forebrain neurons.

**Figure 2:**
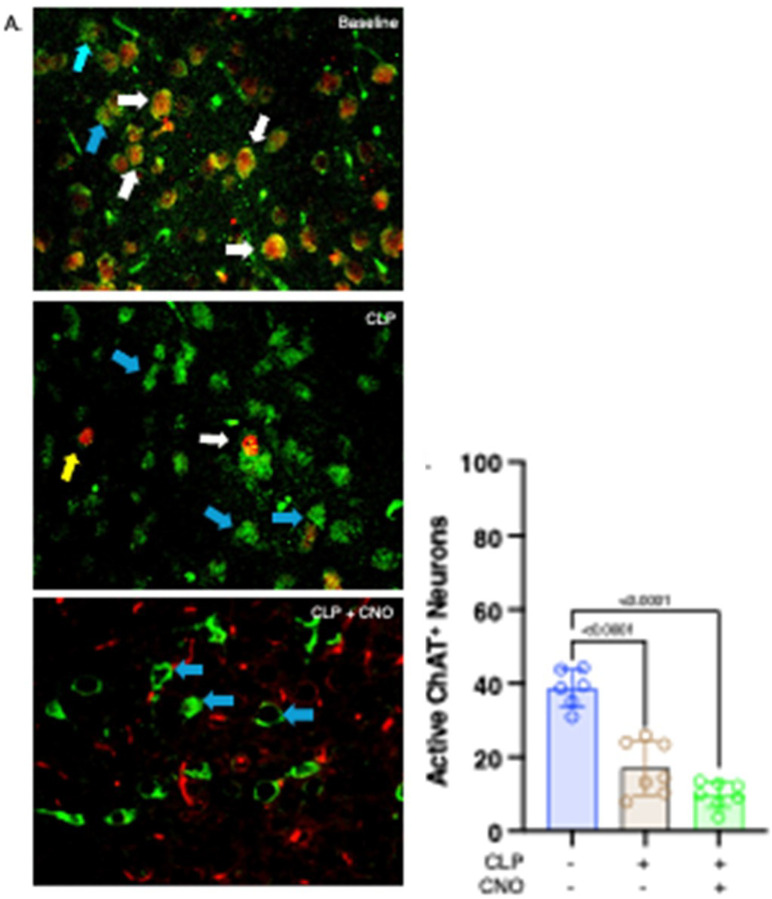
Effects of enhanced orexinergic activity on post - CLP ChAT expression in basal forebrain neurons. Data obtained at baseline in WT mice and at 48 hrs. post-CLP in 1) WT mice and 2) transgenic mice with orexinergic neurons that were chemogenetically activated by CNO administration at 47 hrs. post-CLP. **A. Representative stained sections.** Green stain (blue arrow) – ChAT+. Red stain (yellow arrow) - c-fos +. White arrows - both orexin and c-fos +. **B. Quantification of ChAT activity 48 hrs. post - CLP**. Data measured on 10 non-contiguous 40x-powered fields/slide, 1–2 slides/animal. Data from each animal represented by a circle: Blue – baseline. Brown – CLP. Green – CLP + CNO. Lines; Mean ± SD. One-way ANOVA. P-values noted above each significant comparison.

Demonstrating that activity in ChAT-expressing basal forebrain neurons was lower post - CLP than at baseline (14) in concert with known bi-directional neural connections (28–30) suggests that decreased activation by the orexinergic system contributes to lower activity in ChAT-expressing basal forebrain neurons. We therefore performed CLP on transgenic mice whose orexinergic neurons contained a Designer Receptors Exclusively Activated by Designer Drugs (DREADD) and, by administering clozapine-N-oxide (CNO), chemogenetically activated the orexinergic system at 48 hrs. post – CLP. Activity in ChAT- basal forebrain ChAT-expressing neurons (Figure 2A - representative images; Figure 2B - quantification at 48hrs post-CLP) was significantly lower than activity at baseline, as noted previously (14). Activity following CNO was also significantly lower than baseline and was not statistically distinguishable from findings in untreated post-CLP mice (Figure 2B). Therefore, the low levels of activity in ChAT-expressing basal forebrain neurons that followed CLP did not result from a decrease in orexinergic activity.

### A Loss of M1mAChR-mediated orexinergic activity contributes to high serum levels of TNFa and IL-1b following CLP

**Figure 3:**
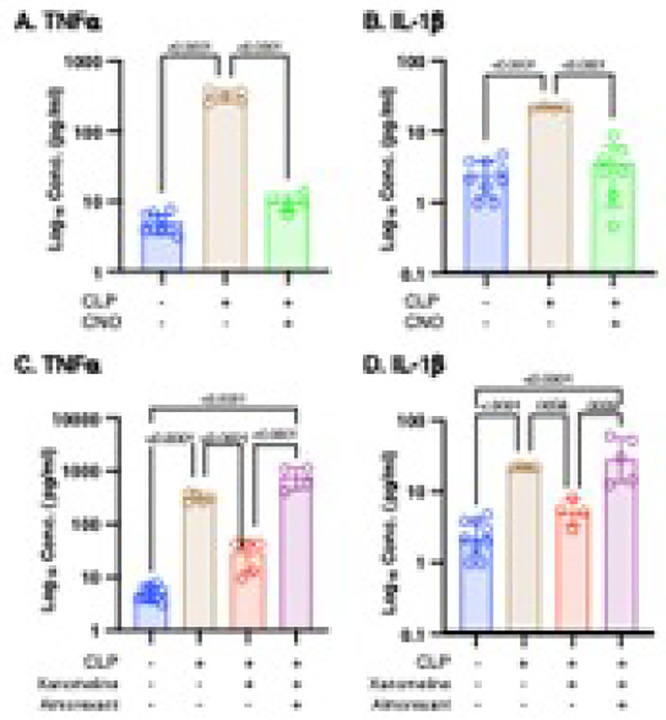
Effects of enhanced orexinergic activity, xanomeline treatment and treatment with xanomeline and almorexant on post - CLP serum levels of TNFa and IL-1 Data at baseline in WT mice (Blue on figure) and at 48 hrs. post-CLP in 1) WT mice (Brown), 2) transgenic mice with orexinergic neurons that were activated chemogenetically by intra-peritoneal (IP) CNO (5mg/kg) adminis-tration at 47 hrs. post CLP (Green), 3) WT mice administered IP xanomeline (5mg/kg)) at the time of CLP and at 23 and 46 hrs post-CLP (Red) and 4) WT mice administered xanomeline + almorexant (50mg/kg) at 23 and 46 hrs post-CLP (Purple). Levels determined using multiplex ELISA (Eve Technologies, Calgary, Alberta, Canada). Each individual animal represented by a circle: Lines; Mean ± SD. Y-axis in log_10_ scale. Samples at baseline and for CLP same as those used in Figure 4. P values noted above each significant comparison. **A. Effects of orexinergic re-activation on post-CLP serum TNFa levels**. One – way ANOVA of log transformed data. **B. Effects of orexinergic re-activation on post-CLP serum IL-1b levels.** One – way ANOVA. **C. Effects of xanomeline on post-CLP serum TNFa levels**. One – way ANOVA of log transformed data. **D. Effects of xanomeline + almorexant on post-CLP serum IL-1b levels.** One – way ANOVA of log transformed data.

**Figure 4:**
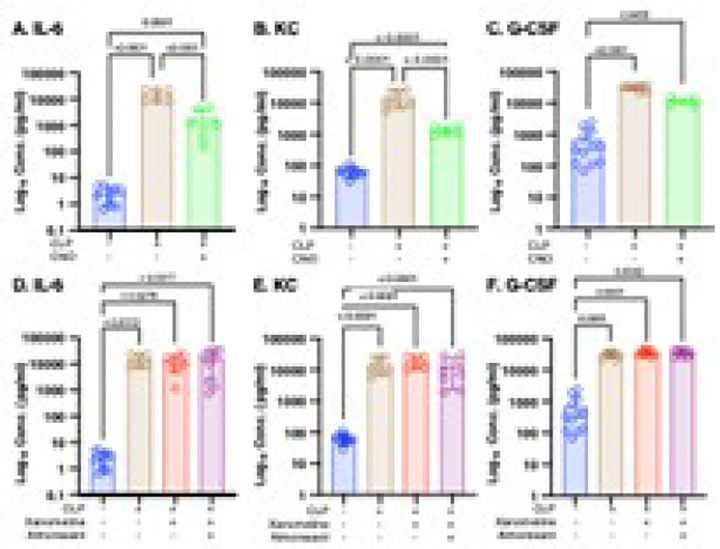
Effects of xanomeline and almorexant on post-CLP serum G-CSF levels. Kruskal-Wallis test. **Effects of enhanced orexinergic activity, xanomeline treatment and treatment with xanomeline and almorexant on post - CLP serum levels of IL-6, KC and G-CSF.** Data at baseline in WT mice (Blue) and at 48 hrs. post-CLP in 1) WT mice (Brown), 2) transgenic mice with orexinergic neurons that were chemogenetically activated by intraperitoneal (IP) CNO (5mg/kg) administration at 47 hrs. post CLP (Green), 3) WT mice administered IP xanomeline (5mg/kg)) at the time of CLP and at 23 and 46 hrs post-CLP (Red) and 4) WT mice administered xanomeline + almorexant (50mg/kg) at 23 and 46 hrs. post-CLP (Purple). Levels determined by multiplex ELISA (Eve Technologies, Calgary, Alberta, Canada). Each animal represented by a circle: Lines; Mean ± SD. Y-axis in log_10_ scale. Samples at baseline and for CLP same as those used in Figure 3. P values noted above each significant comparison **A. Effects of orexinergic re-activation on post-CLP serum IL-6 levels**. **O**ne – way ANOVA of log transformed data. **B. Effects of orexinergic re-activation on post-CLP serum KC levels**. Kruskal-Wallis test. **C. Effects of orexinergic re-activation on post-CLP serum G-CSF levels**. Kruskal-Wallis test. **D. Effects of xanomeline and almorexant on post-CLP serum IL-6 levels**. One – way ANOVA. **E. Effects of xanomeline and almorexant on post-CLP serum KC levels**. One – way ANOVA of log transformed data

Previous studies demonstrated that the mechanism underlying the effects of CLP or LPS on serum concentrations of TNFα and IL-1b (13, 14) was mediated by lower levels of M1mAChR-stimulated activity in the brain (32, 33). Demonstration that the CLP-induced decrease in orexinergic activity and the effects of this reduction on T, HR, RR and hormone (Figure 1) was also mediated, in part, by decreased M1mAChR stimulation led us to examine the contribution of the same mechanism on post-CLP levels of cytokines/chemokines. Post-CLP levels of TNFa, IL-1b, IL-6, KC and G-CSF were higher than baseline levels (Figures 3, 4) (13, 14). Eliminating CLP-induced reductions in orexinergic activity attenuated the high levels of TNFa (Figure 3A) and IL-1b (Figure 3B) noted at 48 hrs. post-CLP. As reported previously (14), xanomeline administration to post-CLP mice led to levels of TNFa (Figure 3C) and IL-1b (Figure 3D) were significantly lower than in untreated animals although levels of TNFa remained significantly higher than those noted at baseline. Following xanomeline administration with the non-specific orexin receptor antagonist almorexant reversed the effects of xanomeline on these cytokines (Figure 3C,D) to levels indistinguishable from those seen following untreated CLP. Thus. One mechanism contributing to high levels of TNFa and IL-1b post-CLP is a loss of M1mAChR stimulation of orexinergic neurons.

### High post-CLP levels of IL-6 and KC result, in part, from a loss of orexinergic activity that is not M1mAChR-mediated.

Following chemogenetic restoration of orexinergic activity post-CLP levels of the cytokine IL-6 (Figure 4A) and the chemokine KC (Figure 4B) were significantly lower than those in untreated animals but were still significantly higher than those noted pre-CLP. Post-CLP administration of xanomeline had no effect on serum levels of IL-6 (Figure 4D) or KC (Figure 4E), confirming previously reported results (14). Thus the mechanism by which CLP increases serum levels of IL-6 and KC involves a loss of orexinergic activity that is independent of M1mAChR stimulation.

### High post-CLP levels of G-CSF result from a mechanism that involves loss of neither orexinergic activity or of M1mAChR stimulation.

As in prior studies (14), levels of G-CSF at 48 hrs. post-CLP were higher than baseline (Figure 4C,F). Chemogenetic re-activation of the orexinergic system had no effect; levels remained significantly different than those noted at baseline and indistinguishable from concentrations in animals not receiving CNO (Figure 4C). As noted previously (14), xanomeline also did not significantly affect post-CLP G-CSF levels (Figure 4F). Therefore, high levels of G-CSF not post-CLP resulted from a mechanism that was independent of the loss either orexinergic activity or a decrease in M1mAChR stimulation.

### Losses of orexinergic activity and of an orexin-independent M1mAChR-mediated response contribute to high post-CLP numbers of splenic macrophages and monocyte-derived dendritic cells (DCs)

**Figure 5:**
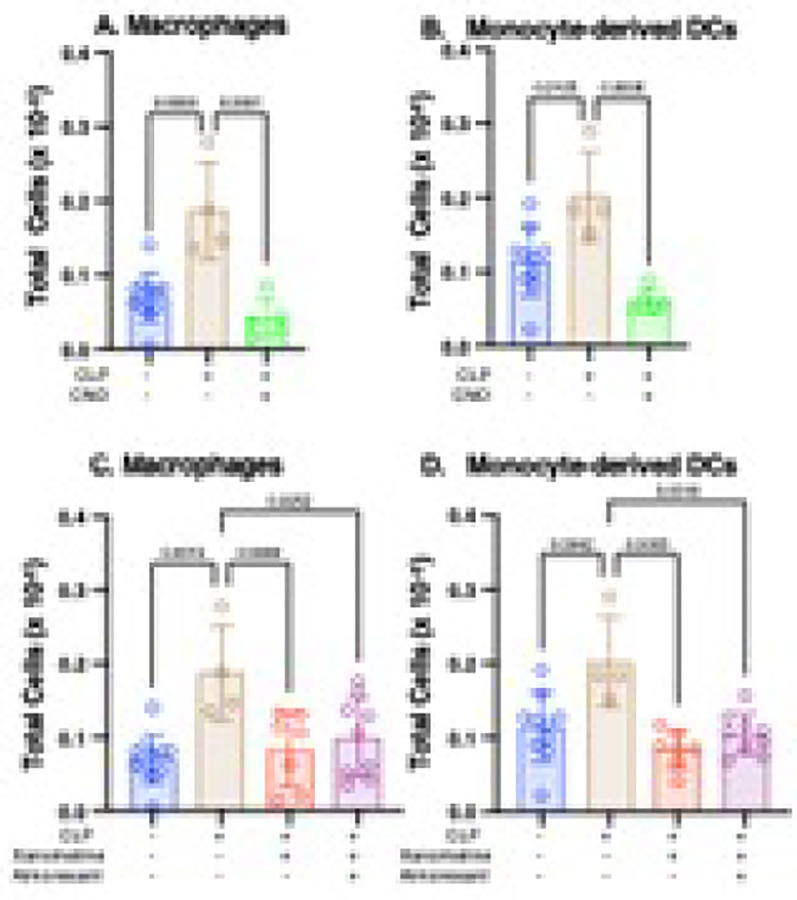
Effects of enhanced orexinergic activity, xanomeline treatment and treatment with xanomeline + almorexant on post - CLP splenic cell subsets. Data obtained at baseline in WT mice (Blue) and at 48 hrs. post-CLP in 1) WT mice (Brown) and 2) transgenic mice with orexinergic neurons that were chemogenetically activated by CNO administration at 47 hrs. post CLP (Green). 3) WT mice administered IP xanomeline (5mg/kg)) at the time of CLP and at 23 and 46 hrs post-CLP (Red) and 4) WT mice administered xanomeline and almorexant (50mg/kg) at 23 and 46 hrs post-CLP (Purple). Quantification by flow cytometry. Each individual animal represented by a circle: Lines; Mean ± SD. Baseline and CLP samples same as those used in Figure 6. P values noted above each significant comparison. **NB**: some, but not all, of the data that was used to determine baseline, post-CLP and post CLP + xanomeline counts of both cell types has been used in a previously published paper (14). **A. Macrophages**. One – way ANOVA. **B. Monocyte Derived Dendritic Cells (DCs).** One–way ANOVA. **C. Macrophages**. One – way ANOVA. **D. Monocyte Derived Dendritic Cells (DCs).** One – way ANOVA.

**Figure 6:**
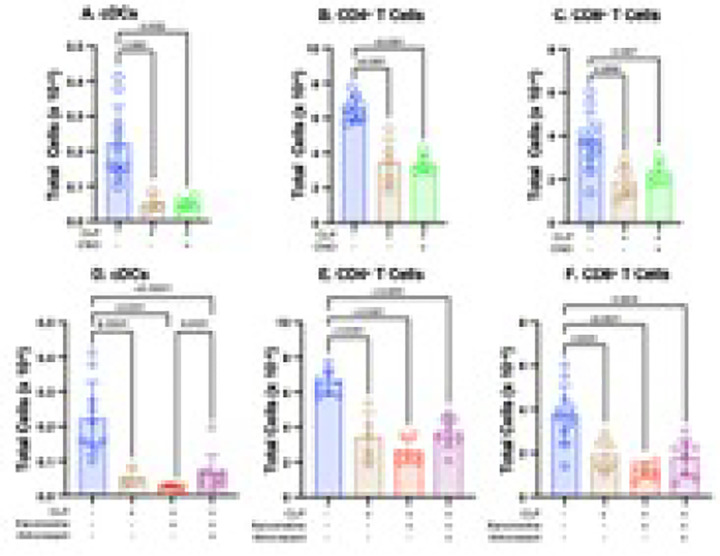
CD8+ T cells. One – way ANOVA **Effects of enhanced orexinergic activity, treatment with xanomeline and treatment with xanomeline + almorexant on post - CLP splenic cell subsets.** Data obtained at baseline in WT mice (Blue) and at 48 hrs. post-CLP in 1) WT mice (Brown), 2) transgenic mice with orexinergic neurons that were chemogenetically activated by CNO administration at 47 hrs. post CLP (Green), 3) WT mice administered IP xanomeline (5mg/kg) at the time of CLP and at 23 and 46 hrs. post-CLP (Red) and 4) WT mice administered xanomeline and almorexant (50mg/kg) at 23 and 46 hrs. post-CLP (Purple). Quantification by flow cytometry. Each animal represented by a circle. Lines; Mean ± SD. P values noted above each significant comparison. Quantification by flow cytometry. **NB**: some, but not all, of the data used to determine baseline, post-CLP and post CLP + xanomeline counts for all three cell types have been used in a previously published paper (14). **A. Central DCs**. One – way ANOVA of log-transformed data. **B. CD4**^**+**^ **T cells**. One – way ANOVA. **C. CD8**^**+**^ **T cells**. One – way ANOVA **D. Central DCs**. One – way ANOVA of log-transformed data. **E. CD4**^**+**^ **T cells**. One – way ANOVA.

We recently demonstrated that CLP increased the numbers of splenic macrophages and inflammatory monocytes via an M1mAChR-dependent mechanism. In contrast, decreases in the numbers of central DCs (cDCs), and CD4^+^ and CD8^+^ T cells did not involve M1 signaling (14). Figures 5 and 6 recapitulate previous findings (14). Following chemogenetic restoration of the CLP-induced reduction in orexinergic activity the numbers of macrophages (Figure 5A) and monocyte-derived DCs (Figure 5B) were significantly lower than in untreated mice and could not be distinguished from baseline levels. As reported previously, the CLP-induced increase in the number of splenic macrophages and inflammatory (monocyte derived) DCs was attenuated by xanomeline treatment (Figures 5C,D)(14). Providing almorexant to xanomeline-treated post-CLP mice did not effect splenic subset cell numbers (Figures 5C,D). Thus, CLP alters the numbers of splenic macrophages and monocyte-derived via a mechanism that includes a loss of orexinergic activity and of an M1mAChR-mediated but orexin-independent process.

### CLP decreases numbers of central DCs (cDCs), CD4^+^ T cells and CD8^+^ T cells by mechanisms that involve neither loss of orexinergic activity or decreased M1mAChR- mediated responses

The data presented above indicated that both lower levels of orexinergic activity and a decrease in an M1mAChR-mediated response independently contributed to the CLP-induced increase in macrophages and monocyte-derived DC numbers. At 48hrs. post-CLP, numbers of splenic cDCs, CD4^+^ T cells and CD8^+^ T cells were lower than at baseline; chemogenetic restoration of orexinergic activity did not affect these findings (Figure 6A–C). As in the previous study (14), xanomeline also had no discernable effect on CLP-induced decreases in cDCs, CD4^+^ T cells and CD8^+^ T cells. Therefore, the mechanism that resulted in a post-CLP decrease in cDCs, CD4^+^ T cells and CD8^+^ T cells involved neither the orexinergic system nor M1mAChR-mediated stimulation.

### CLP increases TNFa and IL-1b production by innate immunes via a mechanism that includes a loss of orexinergic activity.

**Figure 7:**
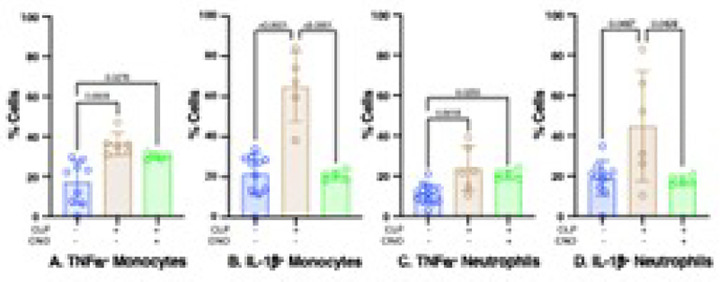
Effects of Orexinergic activation on percentage of post - CLP splenic innate immune cell subsets that expressed cytokines in response to LPS stimulation. Data obtained at baseline in WT mice (Blue) and at 48 hrs. post-CLP in WT mice (Brown) and in transgenic mice with orexinergic neurons that were chemogenetically activated by CNO administration at 47 hrs. post-CLP (Green). Numbers quantified using flow cytometry. Each individual animal represented by a circle: Lines; Mean ± SD. Data at baseline and for CLP same as that used in Figure 8. P values noted above each significant comparison. **NB**: some, but not all, of the data that was used to determine baseline, post-CLP and post CLP + xanomeline counts of both cell types were used in a previously published paper (14). **A. TNFa**^**+**^ **Monocytes**. One – way ANOVA. **B. IL-1b**^**+**^ **Monocytes.** One – way ANOVA. **C. TNFa**^**+**^ **Neutrophils (PMNs)**. One – way ANOVA. **D. IL-1b** ^**+**^ **Neutrophils (PMNs)**. One – way ANOVA of log-transformed data

Low levels of orexinergic activity contribute to CLP-induced effects on serum levels of TNFa and IL-1b (Figure 3) and on the numbers of splenic macrophages (Figure 5). LPS - stimulated innate immune cells express these and other cytokines; we previously showed that CLP high levels of TNFa and IL-1b expression in harvested splenic monocytes and neutrophils stimulated with LPS *ex vivo* (14). Chemogenetic restoration of orexinergic activity in post-CLP mice eliminated the enhanced *ex vivo* expression of IL-1b in response to LPS (Figures 7B,D); the response was indistinguishable from that noted at baseline. Restored orexinergic activity did not, however, affect LPS-stimulated expression of TNFa from either cell type (Figures 7A,C); LPS-stimulated expression remained different than at baseline and in distinguishable from that observed in post-CLP mice with low orexinergic activity. Thus, a loss of orexinergic activity contributed to the elevated expression of IL-1b in both inflammatory monocytes and neutrophils. In contrast, this mechanism did not contribute to the high serum levels of TNFa noted post-CLP.

### CLP increases neutrophil TNFa production via a mechanism that includes a loss of M1mAChR-mediated activity.

**Figure 8:**
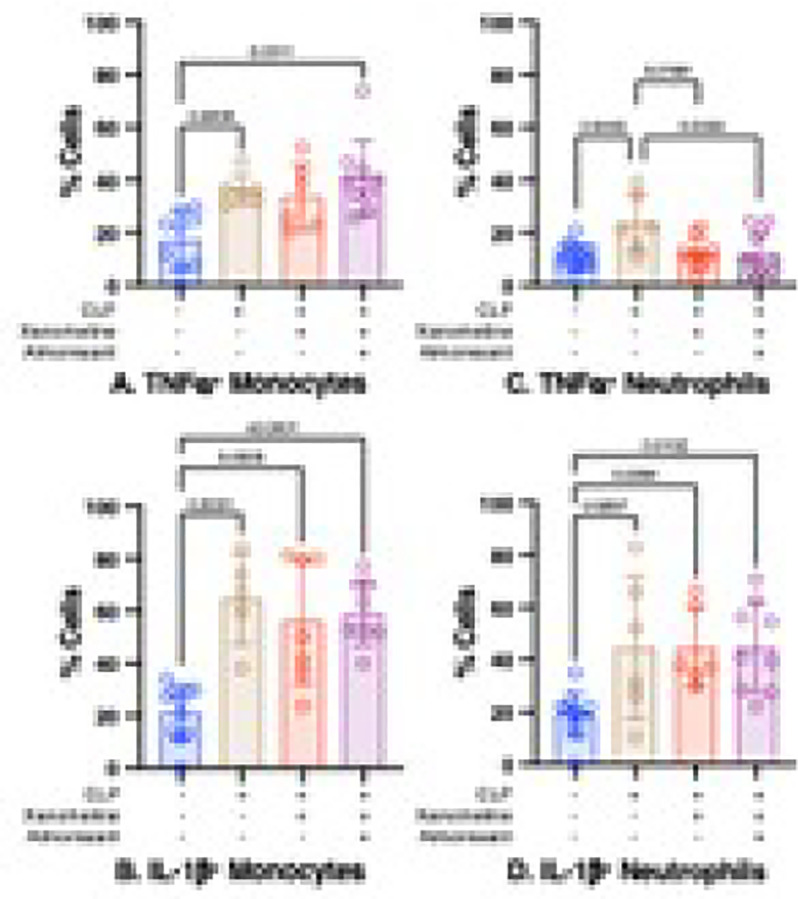
Effects of xanomeline and of xanomeline + almorexant treatment on percentage of post - CLP splenic innate immune cell subsets that expressed cytokines in response to LPS stimulation. Data obtained at baseline in WT mice (Blue) and at 48 hrs. post-CLP in 1) WT mice (Brown) and 2) WT mice administered IP xanomeline (5mg/kg) at the time of CLP and at 23 and 46 hrs. post-CLP (Red) and 3) WT mice administered xanomeline and almorexant (50mg/kg) at 23 and 46 hrs. post-CLP (Purple). Numbers quantified using flow cytometry. Each animal represented by a circle: Lines; Mean ± SD. Data at baseline and for CLP same as that used in Figure 7. P values noted above each significant comparison. **NB**: some, but not all, of the data that was used to determine baseline, post-CLP and post CLP + xanomeline counts of both cell types has been used in a previously published paper (14). **A. TNFa**^**+**^ **Monocytes**. One – way ANOVA. **B. IL-1b**^**+**^ **Monocytes.** One – way ANOVA. **C. TNFa**^**+**^ **Neutrophils**. One – way ANOVA. **D. IL-1b** ^**+**^ **Neutrophils**. One – way ANOVA

We previously demonstrated that xanomeline attenuated CLP-induced increases in the numbers (but not the percentage) of splenic neutrophils and monocytes with elevated TNFa and IL-1b expression following *ex vivo* stimulation with LPS (14). A loss of M1mAChR-mediated orexinergic activity contributed to high post-CLP serum levels of TNFa and IL-1b (Figure 3). Xanomeline reduced the percentage of neutrophils that expressed TNFa (Figure 8C) but had no effect on the IL-1b in neutrophils (Figure 8D) or on either cytokine in monocytes (Figure 8B,C). Adding almorexant to post-CLP mice treated with xanomeline did not affect the percentage of neutrophils expressing TNFa. These data indicate that, while a decrease in an M1mAChR-mediated process contributed to the high LPS-induced TNFa expression in splenic neutrophils isolated post-CLP, that change did not involve M1mAChR-mediated stimulation of orexinergic activity.

## Discussion

In this report we studied how interactions between orexinergic - and/or M1mAChR-mediated responses affect the immune response to CLP. These findings add to our understanding of sepsis by higlighting the impact of altered interactions between the neural and immune systems. More importantly, these data emphasize the key, and perhaps primary, contribution of altered activity in the brain to the pathogenesis of the syndrome-defining dysregulated host response.

The data presented in this paper expand upon prior studies suggesting that the mechanism underlying CLP-induced abnormalities in vital signs and pituitary hormones levels includes a decrease in orexinergic activity (6). Additional examples of the orexinergic contribution to sepsis pathobiology emerge for our data. Specifically, we found that decreased orexinergic activity directly contributes to the CLP-induced increase in the expression of cytokines and chemokines (Figs. 3 and 4) and in alterations in the numbers (Fig. 5) and activity (Fig. 7) of splenic innate immune cells. These data also indicated that some, but not all, of these alterations result from a CLP-induced decrease in M1mAChR-induced stimulation of orexinergic neurons (Fig. 3). Additional CLP-induced differences reflect loss of orexin-dependent but M1mAChR-independent processes (Figs. 4 and 7). Still other effects of CLP are mediated by the M1mAChR but do not involve the orexinergic system (Figs. 5 and 8) (14). (13, 14, 34). These findings highlight the importance of lost orexinergic signaling in sepsis pathobiology.

This study is among the first to demonstrate that altered neuronal activity and a change in the interaction between two specific neural systems in the brain contribute to the pathobiology of sepsis. Previous studies demonstrated that a reduction in orexinergic activity (6) and in M1mAChR-mediated responses (13, 14, 32, 35, 36) are major mechanistic components of both inflammation and sepsis. Inflammation is an adaptive, regulated and tightly coordinated process that is ultimately self-limited. In contrast, sepsis is defined by organ dysfunction and a dysregulated host response (1). One characteristic distinction between the two responses is a loss of communication between different cells and organs, a defect that results from mal-adaptive immune, endocrine and neuronal activity (3). Because investigation into sepsis pathobiology has long focused on activity in the immune and endocrine systems, virtually all clinical interventions have targeted these two systems (37–39). While changes in specific function have been noted, particularly with respect to endocrine support of the cardiovascular system, effects on global outcomes are at best capricious (5, 38, 40). In contrast, exploration of the contribution of the nervous system, and particularly the brain, to sepsis pathobiology has unfortunately lagged despite studies showing that the CNS directly affects both endocrine and immune responses. Changes in neuronal activity can affect organ function more rapidly than the immune or endocrine systems. Thus, our demonstration that pituitary hormone and cytokine/chemokine concentrations were similar to baseline when we revered the effects of CLP on orexinergic and of M1AChR-mediated activity (Figs. 3 and 4) suggest that the brain is the primary driver of the dysregulated host response that defines sepsis.

Exploring orexinergic activity arose from our recognition that this small nidus of neurons was capable of affecting systemic and organ-specific processes that became abnormal in sepsis. By extension, the pathobiological role of altered orexinergic activity in sepsis likely extends beyond the effects on immune function demonstrated here. Our findings here and previously (6) suggest that a loss of orexinergic activity contributes to sepsis-induced metabolic, cardiovascular, pulmonary and endocrine dysfunction. The orexinergic system is known to contributes to pathologic alterations in the respiratory (19, 41), cardiovascular (17, 18, 42, 43), gastrointestinal (44) and renal (45) function. The direct role of decreased orexinergic activity on organ dysfunction post-CLP is currently under investigation.

Several factors led us to posit that CLP reduced M1mAChR stimulation of orexinergic activity. Previous work revealed that M1mAChR-mediated responses arising in brain areas innervated by the basal forebrain cholinergic system contribute to the efferent vagus nerve - mediated anti-inflammatory activity (32). These effects include an interaction between ACh-responsive T cells and innate immune cells in the spleen (35, 46). A loss of central M1mAChR- mediated activity also played a key role in reversal of LPS-induced inflammation in rats or mice (13, 32, 47, 48) by the central M1 agonist xanomeline; this agent also improved survival from CLP (13). Our initial orexinergic work (6) was enhanced by recent studies demonstrate that intranasal injection of orexin A following CLP improved survival and behavior, reduced brain and systemic inflammation and decreased ultrastructural damage in different regions of the brain (49, 50). Finally, identification of bi-directional neuro-anatomic and electrophysiologic connections between basal forebrain cholinergic centers and the orexinergic system of the hypothalamus (28–31) suggested that a change in the interaction between the two systems contributed to the dysregulated host response underlying sepsis pathobiology.

The findings detailed here indicate that CLP-induced abnormalities in T, HR, RR, pituitary hormone levels and serum levels of TNFa and IL-1b reflect decreased stimulation of orexinergic neurons by M1mAChRs. The effects on these two cytokines are consistent with most, but not all (51), reports from other studies independently examining either orexinergic or M1mAChR-mediated activity (13, 32, 47–49, 52–55). Our data provide clear evidence that M1mAChRs modulate orexinergic activity as well as other elements of sepsis. However, the high levels of IL-6 and KC noted post-CLP appear to result from a loss of orexinergic activity that is not M1mAChR-mediated (Fig. 4). CLP-induced effects on numbers of and, to a lesser degree, cytokine elaboration by splenic macrophages and monocyte-derived DCs also result from lower orexinergic and M1mAChR-mediated activity (Figs. 5, 7) but do not involve an interaction between the two. Interestingly, high levels of G-CSF do not appear to result from a loss of either orexinergic activity or of M1mAChR stimulation (Fig. 4); CLP-induced decreases in numbers of splenic CD4^+^ and CD8^+^ T cells and cDCs also arise from a change that is independent of either orexin or M1mAChRs (Fig. 6, 8). These findings highlight the potential importance of non-cholinergic/non-orexinergic neural systems in inflammation/sepsis. Indeed, ketamine, esketamine, and memantine, antagonists of the excitatory neurotransmitter N-methyl-D-aspartate (NMDA), reduced inflammation/sepsis - induced neuronal damage and improved cognition by inhibiting activation of microglial-mediated neuroinflammation (56, 57). Similarly, a loss of b-adrenergic activity has been implicated in the pathogenesis of sepsis-induced encephalopathy; b stimulation attenuated sepsis-induced activation of microglia and/or astrocytes activation and reduced glutamate-mediated toxicity in the hippocampus (58, 59). Altered serotonergic neurotransmission may contribute to cognitive dysfunction following CLP (60). Thus, the effects of systems in the brain that do not involve either orexin or acetylcholine may be highly relevant and should be explored.

Demonstration of a lack of cholinergic or orexinergic activity in some aspects of sepsis pathobiology has additional diagnostic and therapeutic implications. Changes in serum cytokine and chemokine levels have been touted as biomarkers for sepsis; some have been used to delineate specific sepsis sub-phenotypes (61–63) and to quantify vagus-mediated attenuation of inflammation (12, 13, 32, 34, 64). Data indicating that different neural pathways affect serum levels of cytokines and/or chemokines either independently or in concert with each other highlight the complex nature of both cytokine biology and sepsis pathobiology. The multi-faceted biology of sepsis is consistent with the lack of efficacy in directed therapies. Indeed, failed attempts to alter sepsis pathobiology serve as a cautionary tale vis-à-vis the wisdom of intervening in a poorly understood disorder.

The data presented do, however, have therapeutic implications. First, a recent systematic review and meta-analysis examined the use of orexin receptor antagonists in the management of delirium in hospitalized adults (65). This study did not directly examine patients with sepsis, although it did evaluate ICU vs. non-ICU status. The findings detailed here, in concert with our previous work (6) and studies performed by others (49, 50) that addressed the diagnosis of sepsis directly, suggest that further compromising orexinergic activity with receptor antagonists would be detrimental. Conversely, while enhancing orexinergic activity may have value, this approach may be therapeutically problematic. Orexin itself crosses the blood – brain barrier poorly and parenterally-administered orexin is preferentially bound to peripheral orexin receptors (66). Intranasal administration of orexin has been used to alter several aspects of CLP-induced pathobiology in mice (49, 50) and may therefore have clinical promise. But perhaps the greatest concern with direct administration of orexin is the vast array of other neural systems that interact with orexinergic neurons (67). Indeed, the wide distribution of orexin receptors in both the brain and in the periphery suggests that our knowledge of the full extent of orexinergic activity is limited; administration of the drug will almost certainly have unexpected effects.

While enhancing orexinergic activity in patients may be problematic, the same is not true regarding the clinical use of xanomeline or another M1 agonists. Drugs that enhance muscarinic activity have long been used to treat ileus, urinary retention, glaucoma, and many other disorders. Indeed, active investigations are assessing the therapeutic value of centrally acting M1mAChR agonists to treat cognitive and neuropsychiatric dysfunction in disorders such as Alzheimer’s disease (68–70) and schizophrenia (71). The FDA recently approved xanomeline, in combination with a peripherally acting mAChR antagonist (to counter activity outside the brain), for treatment of schizophrenia (72–76). Thus, administration of M1mAChR agonists such as xanomeline may represent a clinically-viable approach to enhancing brain orexinergic activity in sepsis.

The study presented here has several important limitations. Our data examine only a single point in the time course of a dynamic disorder. Previous work has indicated that orexinergic activity post-CLP continues to decline over the course of the disorder (6), which will almost certainly have pathobiological ramifications. Similarly, while M1mAChR-mediated orexinergic enhancement reduced serum levels of TNFa and IL-1b, elevated levels of other cytokines believed to play a key role in sepsis pathobiology (eg, IL-6) respond to orexin via a mechanism that is M1mAChR independent. Thus, use of either xanomeline or orexin in sepsis may not have the desired immunologic effect. Additionally, this work, and most other studies, are focused on the ability of neural systems to affect immune responses. One may, however, consider the immune system as one of a number of organs that become dysfunctional in sepsis. The direct effects of lost orexinergic activity on activity in other systems remains to be assessed.

Differences in human and murine responses to perturbations are also germane. Inflammation in both rodents and humans affects cardio-respiratory function and metabolism but these effects present differently in each species. Further caution is engendered by noting that many potential treatments were effective in animal models but failed to improve outcome in human sepsis (77). CLP is the most commonly used animal model of the disorder (78) but the approach may have limited relevance in humans. Finally, the most important caveat may lie in the outcome variables evaluated. The *sine qua non* of sepsis is organ dysfunction (1). Yet organ function is not used as a primary outcome variable in human sepsis trials and is almost never assessed in murine studies, include the one presented here. Future studies of enhancing either M1mAChR- or orexin-mediated responses in sepsis should include examination of effects on these defining components of sepsis.

## Conclusions

In summary, the data presented here indicate that the mechanisms contributing to post-CLP immunological responses include reduced output from the orexinergic nervous system and a loss of M1mAChR-mediated activity. Some pathobiology is mediated by both systems via a decrease in M1mAChR-stimulated orexinergic activity. These findings suggest that the use of M1AChR agonists such as xanomeline may be valuable. However, M1mAChR activity is not restricted to the orexinergic system or even to the brain. Further, orexin affects many other systems and may modulate activity in systems involving other neurotransmitters, both excitatory and inhibitory. Therapeutic approaches to sepsis-induced immunopathology developed in animal models have not translated to the human disorder. Perhaps it is time to shift the focus away from the immune system and onto the brain and neural system.

## Materials and Methods

### Study Design

All animal experiments met ARRIVE guidelines. The study was designed to test the stated hypotheses while limiting the number of mice sacrificed. Data were collected in baseline (T0) and in animals studied 48hrs. post-CLP. This endpoint was chosen based on previous studies demonstrating that, at this time points, animals met criteria for organ dysfunction (79). Previous experience with frequently – measured variables studied indicated that data from 4–5 surviving animals were sufficient to either demonstrate significance or, based on simulated results, to indicate that the yield from sacrificing additional animals was unlikely to affect these calculations. CLP was performed under isoflurane anesthesia using two 22 - gauge punctures. Animals received 50 mL/kg of sterile saline at the end of the CLP procedure and 0.5 mg/kg of imipenim/cilastatin SQ at the end of surgery and at 23 h. post-procedure as previously described (14). Previous experience studies (13, 14) led us to administer xanomeline (5mg/kg in 100 μL saline IP, cat. # 10790, Cayman Chemical, MI, USA) at end of the CLP procedure and at 23 and 47 hrs. post-CLP. Controls received saline alone. Almorexant (50mg/kg in 100 μL of 2% DMSO/25% β-cyclodextrin, cat. #13638, Cayman Chemical, MI, USA) was administered IP at 23 and 47 hrs. post CLP in conjunction with the xanomeline. Based on pilot data, CNO (5mg/kg in saline IP, cat. # 6329, Tocris Bioscience, UK) was administered to DREADD – expressing transgenic mice at 23 and 47 hrs. post-CLP. Mice were euthanized by cervical dislocation or decapitation at T_0_ or at 48hrs. post-CLP. Blood was obtained via cheek bleed prior to euthanasia or by cardiac puncture immediately after sacrifice.

### Creation of Transgenic Mice with a DREADD expressed in Orexinergic Neurons

On a C57BL6 background we created a transgenic mouse whose orexinergic neurons contained a DREADD encoded by the hM3Dq-mCherry cassette (Genoway S.A, Lyon, France). These neurons expressed cell-surface “designer receptors”; interaction of these proteins with CNO triggered burst-like neuronal activation (80). Heterozygous mice were used for all experiments described here.

### Measurement of T, HR and RR

T, HR and RR measurements at baseline (T_0_) were performed under isoflurane anesthesia. Anesthetics were not used when data were collected just prior to the injections at 24hrs post-CLP and just prior to euthanasia. Vital signs were determined using VEVO 3100 Imaging System (Fujifilm VisualSonics, Toronto Canada). VEVO LAB analysis software (Version 3.1.0; Fujifilm VisualSonics, Toronto, ON, Canada).

### Brain Harvesting, Preparation and Staining

Brains were fixed with 4% paraformaldehyde for 24hrs, immersed in 30% sucrose, embedded and sliced to yield 10 μm sections. Hypothalamic sections were treated with primary antibodies to orexin (mouse anti-orexin, 1:500, R&D Systems Biotechne, Minneapolis MN) and c-Fos (rabbit anti-cFos, 1:500, Cell Signaling Technology, Danvers MA). Secondary antibodies used were donkey anti-mouse conjugated to Alexa 488 (green) for orexin and donkey anti-rabbit conjugated to Alexa 594 (red) for c-fos. Images were obtained using a Zeiss model LSM 880 Confocal microscope. The objective lens had an aperture of 0.95; images were obtained at 40X magnification at room temperature in air. Analysis was performed on 10 non-overlapping images per section. Images were acquired by the microscope via 2 regular photomultiplier tubes, a GaAsp detector and Zen Black Acquisition software. Images were processed using Zen Blue software. The percent of activated orexin – producing cells was determined as

Basal forebrain sections were co-immunostained with antibodies to choline acetytransferase (ChAT, goat anti-ChAT, 1:100, Millipore Sigma, Burlington VT) and c-fos. Secondary antibodies were donkey anti-goat conjugated to Alexa 488 for ChAT, donkey anti-rabbit conjugated to Alexa 594 for c-Fos. A modification of the equation above was used to determine the percent of activated cells.

### Measurements of Cytokine and Pituitary Hormone Levels

Levels of TNFα, IL-1β, IL-6, KC, and G-CSF were determined using a custom multiplex ELISA (Eve Technologies, Calgary, Alberta, Canada). Pituitary hormone levels were determined using ELISA. Each measurement was performed twice.

### Leukocyte Isolation

Spleens harvested post-euthanasia were immediately subjected to 30 minutes of digestion with DNAse (100μg/mL) and Collagenase A (1mg/mL) in complete media at 37°C. Cells were passed through a 70μm filter and resuspended. Red blood cells were lysed, white cells were counted using a Countess II Automated Cell Counter (ThermoFisher, Waltham, MA) and spleen cells were analyzed using flow cytometry. A minimum of 2×10^6^ events were analyzed for each sample.

### Cytokine Production Assays

As previously described (81), single cell suspensions were stimulated with LPS (500ng/ml) for 3 hrs. in the presence of Brefeldin A. All stimulation assays were performed alongside an unstimulated control to assess background production.

### Flow Cytometry

Single-cell suspensions were stained for flow cytometric analysis with LIVE/DEAD fixable viability dye (Life Technologies) and the following antibodies: CD90.2, CD8a, CD4, Ly6C, CD11c, Ly6G, MHCII, IL1β, and TNFα for (full antibody details see (14)). All flow cytometric analyses were performed on a BD LSR Fortessa 16-color cell analyzer and analyzed using FlowJo software version 10 (BD Bioscience, San Jose, CA). Gating strategies for innate immune cells can be found in (14). Importantly, some, but not all, of the data that was used to determine baseline, post-CLP and post CLP + xanomeline cell counts has been used in a previously published paper (14).

### Statistics

The residuals of the data were examined using D’Agostino-Pearson omnibus, Anderson-Darling, Shapiro-Wilk and Kolmogorov-Smirnov tests and via Q:Q plots. If residuals were normally distributed, we used one-way ANOVA corrected with Tukey’s multiple comparison test to identify statistical significance. If the residuals were not normally distributed, we log (ln) transformed the data. One-way ANOVA with the Tukey correction was applied if the residuals of the transformed data were normally distributed. If residuals were still not normally distributed, we applied the Krusal-Wallis non-parametric test for significance. In all cases the threshold for significance was set at P < 0.05.

## Data Availability

The data derived from the work described in this manuscript are contained in a Supporting Data Values file and are available on request from the Corresponding Author.

## Abbreviations

ACTH: adrenocorticotropin
cDC: central dendritic cell
ChAT: choline acetyltransferase
CLP: cecal ligation and puncture
CNO: clozapine–N–oxide
DC: dendritic cell
DREADD: Designer Receptor Exclusively Activated by Designer Drugs
GCSF: granulocyte colony stimulating factor
GH: growth hormone
HR: heart rate
ICV: intra–cerebroventricular
IL: interleukin
KC: keratinocyte–derived cytokine, also known as CXCL1
LPS: lipopolysaccharide
M1mAChR: type 1muscarinic acetylcholine receptor
NMDA: N–methyl–D–aspartate
RR: respiratory rate
T: temperature
TNF: tumor necrosis factor
TSH: thyroid stimulating hormone
